# PUSHING BACK AGAINST AI: USING UNGRADING TO PROMOTE CRITICAL THINKING AND INTRINSIC MOTIVATION

**DOI:** 10.1093/geroni/igad104.1082

**Published:** 2023-12-21

**Authors:** Jenny Inker, Catherine MacDonald, Michael Forder, Shannon Arnette, Leland Waters

**Affiliations:** Virginia Commonwealth University, Richmond, Virginia, United States; Virginia Commonwealth University, Richmond, Virginia, United States; Virginia Commonwealth University, Richmond, Virginia, United States; Virginia Commonwealth University, Richmond, Virginia, United States; Virginia Commonwealth University, Richmond, Virginia, United States

## Abstract

Ungrading is an innovative pedagogical practice in which instructors give frequent, detailed feedback on students’ work and/or encourage self-evaluation rather than assigning them points or letter grades. Ungrading has been shown to increase students’ intrinsic motivation to learn, their willingness to take intellectual risks, and the quality of their thinking. Prior to the introduction of advanced artificial intelligence (AI) writing in academia, grades were shown to be a motivating factor for cheating. Ungrading removes the pressure of grades, thus making the use of AI and other services including paper writing and test answer sharing less enticing. Ungrading could be key to pushing back against the potential for the advent of artificial intelligence tools, which have many universities “alarmed.” Educators at Virginia Commonwealth University College of Health Professions, supported by their Director of e-Learning, planned and piloted ungrading in one undergraduate and two graduate gerontology courses. Twenty-five students completed pre and post-surveys asking for their reactions to ungrading, if their views had changed as a result of the ungrading pilot, as well as their perceptions of traditional grading schemes. They also completed scaffolded assignments with multiple opportunities for receiving in-depth instructor feedback, rather than grades, at various points during the semester. Faculty journaled their ungrading experience in order to recognize how it can be a disruptive, and at times, uncomfortable practice and as a result, educators can better guide themselves and students through the transition. This presentation will include an Implementation Guide for gerontology faculty who wish to implement ungrading.

